# Mr. Dunning's Letter to Dr. James Clarke

**Published:** 1806-09

**Authors:** James Clarke

**Affiliations:** Physician to the Nottingham Vaccine Institution. Nottingham


### *To the Editors of the Medical and Physical Journal.*

Gentlemen,

The following Letter is written with such "a spirit of politeness," that I could not but be highly pleased with it, and have no doubt but it will be acceptable to your numerous readers.

The pamphlet to which Mr. Dunning alludes, I had not seen, but I feel much gratified by such coincidence of opinion, drawn from practical facts.

I am, &c.

JAMES CLARKE,

Physician to the Nottingham Vaccine Institution.

*Nottingham, Aug.* 11, 1806.

---

To Dr. JAMES CLARK, *Nottingham.*

SIR,

I have just read your paper in the last Medical Journal, with very great satisfaction; the coincidence of your observations, with those of mine, detailed in the enclosed, which was published and pretty generally circulated in this neighbourhood, more than fifteen months ago, is indeed remarkable; and as I have no reason to believe that you have hitherto seen my paper, the two papers must be considered as remarkably corroborative and illustrative of each other. Reiterated experiments and observations have fully confirmed to me, that the principle which I have insisted on, is not a mere hypothesis, and certainly your valuable Report confirms, almost beyond the possibility of doubting, that my suggestions are founded in Nature and in truth.

A respectable surgeon in this town, lately re-vaccinated four subjects of ruptured vesicle, and he was led to make this experiment from the observations I had printed. In one of the four, the process went through all the stages of vesicle, erythema, induration, and scabbing, yet each of these stages was, perhaps, marked (for I repeatedly saw this experiment,) with some minute shades of difference, from cases of primary vaccination, but vaccinity certainly strongly characterized every part of this inoculation.—In the other three, a slight vesication, desiccation, and desquamation, rapidly succeeded each other, and disappeared in a few days.

Every occurrence of small-pox, after what I have ventured to term incomplete vaccination, has been very mild, and indisputably of a character modified by previous vaccination; and hence I have called these results, secondary variolæ. Allow me to say, that you are entirely at liberty to make what use you please of the enclosed, judging it wholly wholly unnecessary to make an apology for writing on a professional subject, to a Gentleman who appears to me to be animated by the most candid and liberal motives. I have the honour to subscribe myself,

Your most obedient humble servant,

RICHARD DUNNING.

*Plymouth Dock, August* 5, 1806.

